# A Meta-analysis of the Severity of Acute Pancreatitis (AP) in COVID-19 Infection

**DOI:** 10.7759/cureus.38764

**Published:** 2023-05-09

**Authors:** Ahmed Ali Aziz, Muhammad Ali Aziz, Nosheen Omar, Maleeha Saleem, Karan H Pahuja, Muhammad Haseeb ul Rasool, Rehan Shah

**Affiliations:** 1 Internal Medicine, Saint Francis Medical Center, Trenton, USA; 2 Internal Medicine, BronxCare Health System, New York City, USA; 3 Anatomy, University of Health Sciences, Lahore, PAK; 4 Medicine, Icahn School of Medicine at Mount Sinai, Queens Hospital Center, New York, USA

**Keywords:** acute necrotizing pancreatitis, systematic review and meta-analysis, acute pancreatitis, sars-cov-2 infection, covid-19

## Abstract

Many studies have reported severe acute respiratory syndrome coronavirus 2 (SARS-CoV-2) affecting the gastrointestinal tract and causing gastritis, colitis, duodenitis and acute pancreatitis (AP). We conducted a meta-analysis to evaluate if SARS-CoV-2 infection (COVID-19 infection) affects the outcomes and severity of AP. We searched for articles in PubMed (MEDLINE), Cochrane Library, and clinicaltrials.gov databases and included studies comparing the outcomes of AP in patients with and without COVID-19. Our outcomes were the mean age of occurrence of AP, Charlson Comorbidity Index, incidence of idiopathic etiology of AP, severity of AP, incidence of necrotizing pancreatitis, need for intensive care unit (ICU) admission, and mortality between the two cohorts. We included five observational studies with a total population of 2,446 patients. Our results showed that in COVID-19 patients; AP had higher odds of having an idiopathic etiology (odds ratio, OR 3.14, 95% confidence interval, CI 1.36-7.27), be more severe (OR 3.26, 95% CI 1.47-7.49), had higher risk for pancreatic necrosis (OR 2.40, 95% CI 1.62-3.55), require ICU admission (OR 4.28, 95% CI 2.88-6.37) and had higher mortality (OR 5.75, 95% CI 3.62-9.14) than in patients without COVID-19 infection. Our study concluded that SARS-CoV-2 infection does increase the morbidity and mortality associated with AP and further large-scale multi-center studies are needed to confirm these results.

## Introduction and background

The severe acute respiratory syndrome coronavirus 2 (SARS-CoV-2) usually causes respiratory symptoms but, gastrointestinal, hepatic, and pancreatic involvement have been reported as well [1]. Pancreatic injury in SARS-CoV-2 infection (COVID-19 infection) is thought to be secondary to the direct cytotoxic effect of SARS-Cov-2 on pancreatic acinar cells through the angiotensin-converting enzyme 2 (ACE2) receptors on the pancreas which are the main receptors for SARS-CoV-2 [2-4]. We hence conducted a systematic review and meta-analysis to evaluate if SARS-CoV-2 infection affects the severity and outcomes of acute pancreatitis (AP).

## Review

Search strategy

We searched Cochrane Library, clinicaltrials.gov and PubMed (MEDLINE) databases from inception till February 3rd, 2022 for published articles using medical subject headings keywords “COVID-19” OR “SARS-CoV-2” AND “acute pancreatitis.” We followed the Standard Preferred Reporting Items for Systematic reviews and Meta-Analysis (PRISMA) statement and Cochrane guidelines [5-6] for the search process. Studies retrieved by the search strategy were screened independently by two authors (Aziz A and Aziz M) to identify studies that met the predefined inclusion criteria. Any disagreements between the reviewers were resolved through consensus and opinion of other authors. References of eligible studies were searched for any additional articles that might be eligible for our study. Citations were exported to a reference management program (Microsoft Excel 2020, Microsoft® Corp., Redmond, WA).

Eligibility criteria

We included any randomized controlled trials, prospective or retrospective studies that compared outcomes of AP in a COVID-19 negative control group and a COVID-19 positive cohort. We included studies with a patient population age greater than or equal to 18 years including pregnant patients. We only included articles whose full texts were available in English or if an English translation was available. We only included studies involving human subjects.

Results of the search

**Figure 1:**
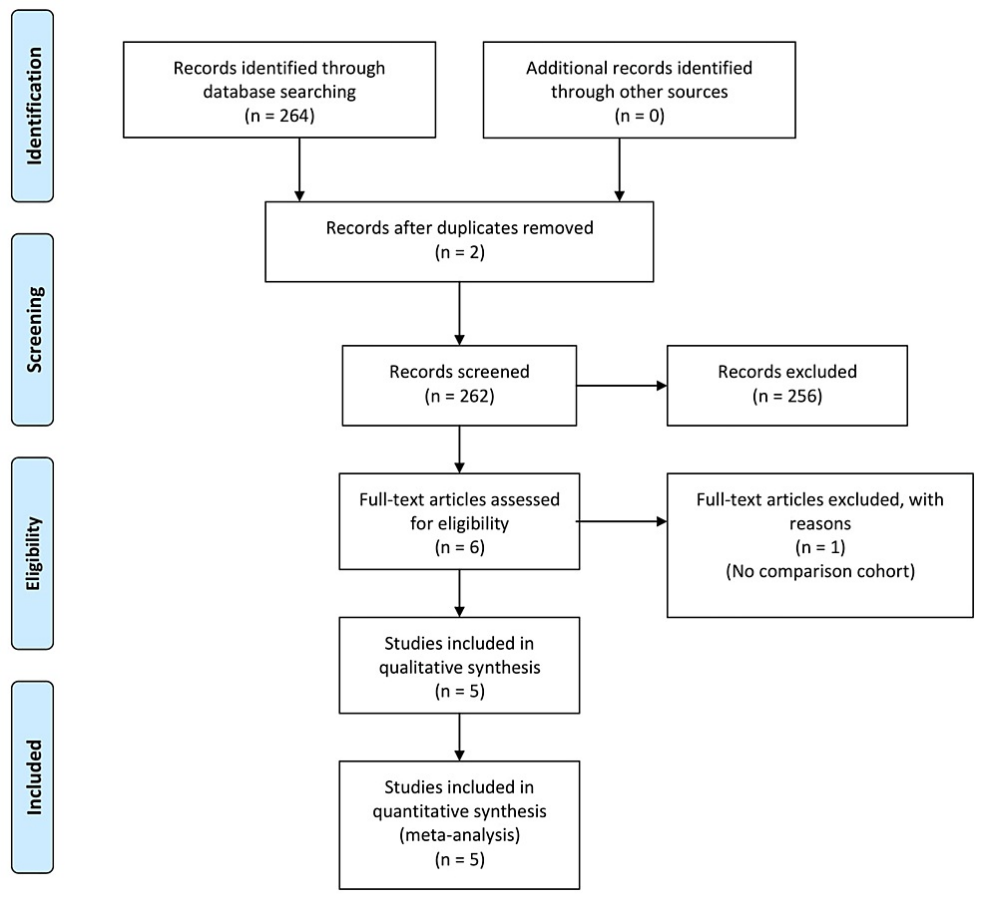
PRISMA flow diagram demonstrating the selection of studies included in the meta-analysis. PRISMA, Preferred Reporting Items for Systematic Reviews and Meta-Analyses

The initial search yielded 264 articles. Two duplicate articles were removed leaving 262 articles. Some 123 articles were excluded on the basis of title read, 133 on abstract read, and one article was excluded on full text read as these did not meet the inclusion criteria of our study (Figure 1). Hence, five articles were included in our study [7-11].

Included studies

A total of five studies including one prospective, multicenter cohort study [11] and four retrospective studies [7-10] were included in the meta-analysis as shown in Table 1.

**Table 1:** Summary of studies included in the meta-analysis. AP, acute pancreatitis; ICU, intensive care unit; MV, mechanical ventilation; RC, retrospective cohort; PMC, prospective multicenter cohort; CCI, Charlson comorbidity index; BISAP, Bedside Index for Severity of Acute Pancreatitis

No.	Reference	Year	Study design	Population no.	Comorbidity assessment	Severity of AP	ICU admission or MV
1	Dirweesh et al. [7]	2020	RC	75	CCI score	BISAP	ICU
2	Inamdar et al. [8]	2020	RC	189	CCI score	BISAP	MV
3	Karaali et al. [9]	2021	RC	189	CCI score	BISAP	ICU
4	Miro et al. [10]	2021	RC	216	Not mentioned	BISAP	ICU
5	Pandanaboyana et al. [11]	2021	PMC	1777	Not mentioned	Persistent organ failure	ICU

Outcome measures

All authors independently extracted data for the outcomes. Later, the data extracted by each individual author was compared and any discrepancies found between the authors were resolved through consensus. We used the RevMan software (Biostat, Englewood, NJ, USA) to analyze the outcomes. The random effects model was used to obtain the final pooled risk estimate. The I2 statistics were calculated to assess heterogeneity between studies. p30% was interpreted as significant heterogeneity. Our outcomes included mean age of occurrence of AP, Charlson Comorbidity index (CCI), incidence of idiopathic etiology of AP, severity of AP, incidence of necrotizing pancreatitis, need for intensive care unit (ICU) admission and mortality between the two cohorts. We used a CCI value of greater than or equal to five to compare the comorbidities present in patient population of both cohorts. The ICU admission was defined as need for ICU transfer or need for mechanical ventilation. Severe AP was defined as either Bedside Index for Severity of Acute Pancreatitis (BISAP) score greater than or equal to three or persistent end organ failure for greater than 48 h. 

Outcome analysis

Age

**Figure 2:**
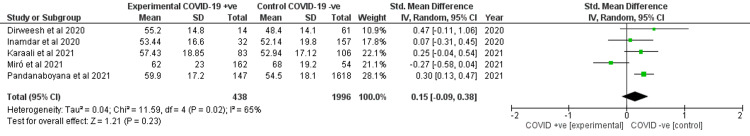
Forest plot for the standard mean difference of the age of occurrence of AP between the two cohorts. Dirweesh et al. (2020) [7], Inamdar et al. (2020) [8], Karaali et al. (2021) [9], Miro et al. (2021) [10], and Pandanaboyana et al. (2021) [11]. AP, acute pancreatitis

There was no statistically significant standard mean difference (SMD) in the age of occurrence of AP in patients with and without COVID-19 infection (SMD 0.15, 95% CI -0.09-0.38). The heterogeneity (I2) was 65% (Figure 2).

Charlson Comorbidity Index

**Figure 3:**
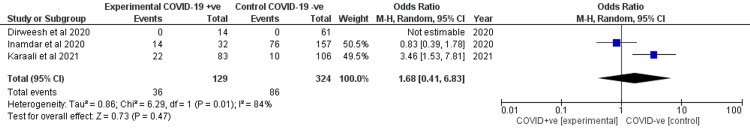
Forest plot for the CCI between the two cohorts. Dirweesh et al. (2020) [7], Inamdar et al. (2020) [8], and Karaali et al. (2021) [9]. CCI, Charlson Comorbidity Index

Three studies reported the CCI. There was no statistically significant difference in the CCI in the two population groups (OR 1.68, 95% CI 0.41-6.83). This is shown in the forest plot in Figure 3. The heterogeneity (I2) was 84%.

Idiopathic etiology of acute pancreatitis

**Figure 4:**
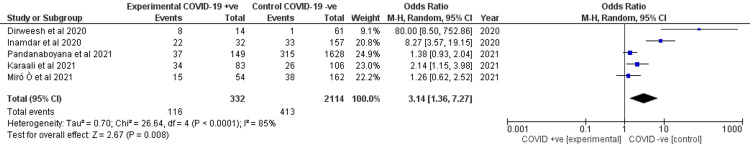
Forest plot for the idiopathic etiology of AP between the two cohorts. Dirweesh et al. (2020) [7], Inamdar et al. (2020) [8], Karaali et al. (2021) [9], Miro et al. (2021) [10], and Pandanaboyana et al. (2021) [11]. AP, acute pancreatitis

Incidence of idiopathic etiology of AP was reported by all studies. COVID-19 was associated with higher odds of having AP with an idiopathic etiology with a statistical significance (OR 3.14, 95% CI 1.36-7.27) as shown in forest plot in Figure 4. There was a high heterogeneity (I2 = 85%).

Severity of acute pancreatitis

**Figure 5:**
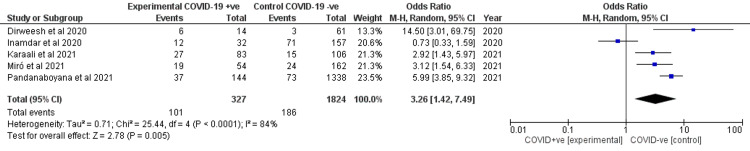
Forest plot for the severity of AP between the two cohorts. Dirweesh et al. (2020) [7], Inamdar et al. (2020) [8], Karaali et al. (2021) [9], Miro et al. (2021) [10], and Pandanaboyana et al. (2021) [11]. AP, acute pancreatitis

Severity of AP was reported by all studies. COVID-19 increased the odds of severe AP (OR 3.26, 95% CI 1.47-7.49) with a statistical significance as shown in Figure 5. There was significant heterogeneity (I2 = 84%).

Incidence of necrotizing pancreatitis

**Figure 6:**
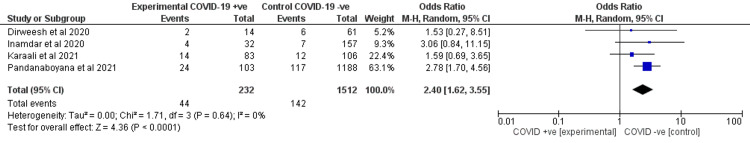
Forest plot for the incidence of necrotizing pancreatitis between the two cohorts. Dirweesh et al. (2020) [7], Inamdar et al. (2020) [8], Karaali et al. (2021) [9], and Pandanaboyana et al. (2021) [11].

The incidence of pancreatic necrosis was reported by four studies. Our analysis showed that patients with COVID-19 infection had statistically significant higher odds of developing necrotizing pancreatitis (OR 2.40, 95% CI 1.62-3.55) as shown in Figure 6. There was no heterogeneity (I2 = 0%). 

Need for ICU admission

**Figure 7:**
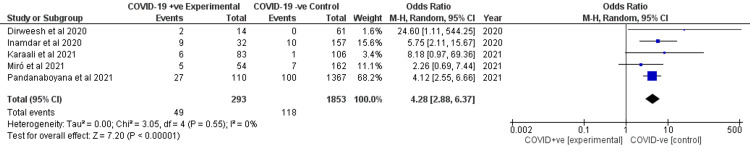
Forest plot for the need for ICU admission between the two cohorts. Dirweesh et al. (2020) [7], Inamdar et al. (2020) [8], Karaali et al. (2021) [9], Miro et al. (2021) [10], and Pandanaboyana et al. (2021) [11]. ICU, intensive care unit

All studies reported the need for ICU admission amongst the two groups. Our analysis showed that AP patients with COVID-19 infection had higher odds of requiring ICU admission (OR 4.28, 95% CI 2.88-6.37) as shown in Figure 7. There was no heterogeneity (I2 = 0%). 

Mortality

**Figure 8:**
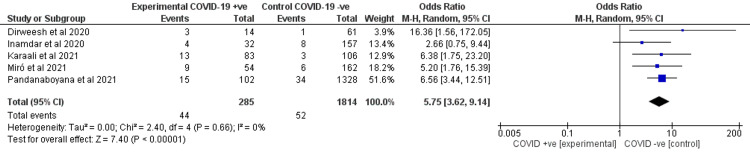
Forest plot for the mortality in AP patients with and without COVID-19 infection. Dirweesh et al. (2020) [7], Inamdar et al. (2020) [8], Karaali et al. (2021) [9], Miro et al. (2021) [10], and Pandanaboyana et al. (2021) [11]. AP, acute pancreatitis

All studies reported the mortality rates between the two cohorts. Our analysis showed that COVID-19 increased mortality (OR 5.75, 95% CI 3.62-9.14) in AP patients with a statistical significance as shown in Figure 8. There was no heterogeneity (I2 = 0%).

Discussion

To our knowledge our study is the second in literature of its kind to investigate the severity of AP in patients with and without COVID-19 infection. We included a total of five studies in this review with a total population of 2,446 patients. Our outcomes were a comparison of mean age of occurrence of AP, CCI, idiopathic etiology of AP, severity of AP, incidence of necrotizing pancreatitis, need for ICU admission and mortality between the two cohorts.

Our analysis shows no statistically significant difference in the mean age of occurrence of AP in patients with and without COVID-19 infection and no significant difference in the CCI between the two groups. This is important as it shows that neither of the cohorts was older or younger or had less or more comorbidities.

Our analysis shows that AP in patients with COVID-19 infection is more severe, has a higher incidence of pancreatic necrosis, is more likely to require ICU admission, has a higher mortality and is more likely to have an idiopathic etiology than in patients without COVID-19 infection.

We noted that in the included studies severity of AP was based on the BISAP score. The BISAP score includes points for pleural effusion and points for systemic inflammatory response syndrome (SIRS) if it is greater than two [12]. Patients with COVID-19 infection were more likely to have pleural effusion due to pneumonia and systemic inflammatory response from COVID-19 infection induced cytokine storm. The elevated SIRS score and presence of pleural effusion in AP patients with COVID-19 infection would increase their measured BISAP scores and hence increased the severity of AP in patients with COVID-19 infection. We also noticed that patients with COVID-19 infection had higher incidence of pancreatic necrosis than patients without COVID-19 infection. This can be explained by the direct cytotoxic effect of the virus on pancreatic acinar cells through the ACE2 receptors and the cytokine storm causing arterial thrombosis and neutrophil extracellular traps which might contribute to the higher incidence of necrotizing pancreatitis in patients with COVID-19 infection [13-15].

Our analysis shows higher odds of requiring ICU admission and mortality in patients with COVID-19 infection. This is likely due to the fact that patients with COVID-19 infection were sicker, had two on going disease processes at the same time, had higher incidence of septic shock and multi-organ dysfunction which would increase their need of ICU admission and mortality. Chiarello et al. reported worse outcomes in AP patients with concomitant COVID-19 induced lung injury [16]. Meng et al. and Li et al. reported that the presence and prevalence of pneumonia increased mortality in patients with COVID-19 [17-18].

We also found that the rate of idiopathic etiology of AP was higher in patients with COVID-19 infection. SARS-CoV-2 might cause AP through direct cytotoxic effect due to its affinity for the ACE2 receptors located on the pancreas or through endothelial damage, thrombosis, or cytokine storm [4, 19-20]. However, a possible confounding variable might be the drugs such as steroids, remdesivir, tocilizumab, and doxycycline used in treating COVID-19 which can themselves cause AP and were not used in patients without COVID-19 infection [21-24]. Several viruses such as cytomegalovirus (CMV), Epstein-Barr virus (EBV), and herpes simplex virus (HSV) have been associated with causing AP and SARS-CoV-2 might be one of them as well [25].

Limitations of our study

Our study has certain limitations. Due to small number of studies included it has a publication bias. The process of data collection and analysis in the included studies was not standardized. The diagnosis of COVID-19 was made by clinical symptoms and imaging findings and not by confirmed polymerase chain reaction (PCR) positive testing in a small number of patients in studies by Pandanaboyana et al. [11] and Miro et al. [10] earlier in the pandemic when routine testing was not available. This could be a source of selection bias in the included studies. 

## Conclusions

Our study shows that AP in patients with COVID-19 infection is more severe with increased morbidity and mortality than in patients without COVID-19 infection. Hence, regardless of age or associated co-morbidities when present together in a patient; prompt and aggressive measures should be taken to treat both AP and COVID-19 infection to reduce the severity of AP, prevent pancreatic necrosis and mortality. Some viruses such as CMV, EBV, and HSV have been associated to cause AP and SARS-Cov-2 might be one of them, however, further larger-scale clinical trials with more population size are needed to confirm or refute this hypothesis.
